# An anti-inflammatory role for C/EBPδ in human brain pericytes

**DOI:** 10.1038/srep12132

**Published:** 2015-07-13

**Authors:** Justin Rustenhoven, Emma L. Scotter, Deidre Jansson, Dan T. Kho, Robyn L. Oldfield, Peter S. Bergin, Edward W. Mee, Richard L. M. Faull, Maurice A. Curtis, Scott E. Graham, Thomas I-H. Park, Mike Dragunow

**Affiliations:** 1 Department of Pharmacology and Clinical Pharmacology; 2 Gravida National Centre for Growth and Development; 3 Department of Anatomy with Radiology; 4 Centre for Brain Research; 5 The University of Auckland, 1023, Auckland, New Zealand; 6 Lab Plus, Auckland City Hospital, 1023, Auckland, New Zealand

## Abstract

Neuroinflammation contributes to the pathogenesis of several neurological disorders and pericytes are implicated in brain inflammatory processes. Cellular inflammatory responses are orchestrated by transcription factors but information on transcriptional control in pericytes is lacking. Because the transcription factor CCAAT/enhancer binding protein delta (C/EBPδ) is induced in a number of inflammatory brain disorders, we sought to investigate its role in regulating pericyte immune responses. Our results reveal that C/EBPδ is induced in a concentration- and time-dependent fashion in human brain pericytes by interleukin-1β (IL-1β). To investigate the function of the induced C/EBPδ in pericytes we used siRNA to knockdown IL-1β-induced C/EBPδ expression. C/EBPδ knockdown enhanced IL-1β-induced production of intracellular adhesion molecule-1 (ICAM-1), interleukin-8, monocyte chemoattractant protein-1 (MCP-1) and IL-1β, whilst attenuating cyclooxygenase-2 and superoxide dismutase-2 gene expression. Altered ICAM-1 and MCP-1 protein expression were confirmed by cytometric bead array and immunocytochemistry. Our results show that knock-down of C/EBPδ expression in pericytes following immune stimulation increased chemokine and adhesion molecule expression, thus modifying the human brain pericyte inflammatory response. The induction of C/EBPδ following immune stimulation may act to limit infiltration of peripheral immune cells, thereby preventing further inflammatory responses in the brain.

Neuroinflammation contributes to the development and progression of epilepsy^1^, traumatic brain injuries^2^, stroke^3^ and many neurodegenerative diseases^4^ such as motor neuron disease and Alzheimer’s disease. Microglia, and to a lesser extent astrocytes, are believed to be the primary initiators of neuroinflammation and can promote neuronal loss through the secretion of neurotoxic molecules^5^,^6^. In addition to brain glia, pericytes also contribute to the inflammatory response^7^,^8^,^9^,^10^. Brain pericytes are situated surrounding and contacting endothelial cells of brain capillaries and together with astrocytes, neurons and microglia form the neurovascular unit^11^. Pericyte vascular coverage is essential for the formation and maintenance of the blood-brain barrier (BBB) and the regulation of cerebral blood flow highlighting its importance in central nervous system (CNS) homeostasis^12^,^13^,^14^.

Like brain glia, pericytes can also respond to a range of immunogenic stimuli to induce pro-inflammatory molecules including cytokines e.g., interleukin-6 and 8 (IL-6 and IL-8), chemokines such as monocyte chemoattractant protein-1 (MCP-1) and interferon gamma-induced protein-10 (IP-10) and adhesion molecules such as intracellular adhesion molecule-1 (ICAM-1) and vascular cell adhesion molecule-1 (VCAM-1)^8^,^9^,^10^. Induction of these mediators can promote peripheral immune cell infiltration^15^,^16^,^17^, as well as local microglial cell migration^18^, proliferation^19^,^20^ and activation^21^ enhancing the pro-inflammatory phenotype of the brain and potentially contributing to neuronal loss.

Cellular inflammatory responses are orchestrated largely through transcription factor mediated gene expression^22^. By virtue of conserved promoter/enhancer DNA sequences, a single transcription factor may regulate the expression of numerous inflammatory genes making them an attractive target for anti-inflammatory interventions. Involvement of the prototypical inflammatory transcription factor nuclear factor-kappa B (NF-kB) has previously been identified in pericyte activation^8^,^10^. However, evidence regarding further transcription factor involvement in pericyte inflammatory responses is currently lacking and warrants further investigation.

A role in mediating pro-inflammatory gene expression has recently been observed with several members of the CCAAT/enhancer binding protein (C/EBP) family of transcription factors, particularly with C/EBPα^23^, C/EBPβ^24^ and C/EBPδ^25^,^26^. C/EBPs are members of the bZIP family of transcription factors and many inflammatory genes include CCAAT binding motifs in their promoter/enhancer region^27^. C/EBP family members require dimerisation for DNA binding and do so by forming homodimers or heterodimers with other C/EBP family members, or associated transcription factors including NF-kB^28^.

In response to inflammatory stimuli many tissues demonstrate induction of C/EBPδ expression. In the Alzheimer’s brain and in spinal cord of amyotrophic lateral sclerosis (ALS) patients, both of which have a significant inflammatory component, enhanced C/EBPδ protein expression has been observed in astrocytes and microglia respectively^26^,^29^. The functional effects of this induction however remain unclear. Studies utilising rodent glia have suggested C/EBPδ has a pro-inflammatory role in the brain through enhancement of inflammatory gene transcription^25^,^26^. Indeed this has also been seen in other tissues, including the liver and lung where attenuation of C/EBPδ expression dampened inflammatory responses^30^,^31^,^32^. However, C/EBPδ induction has also been shown to inhibit pro-inflammatory gene expression in the pancreas^33^ whilst C/EBPδ deficiency enhanced tubulointerstitial fibrosis, a renal condition with an inflammatory component^34^. Furthermore, induction of a closely related family member C/EBPβ has both anti-inflammatory^24^ and pro-inflammatory roles in the brain^35^. As such, it appears C/EBP family members including C/EBPδ act in a cell and context dependant manner allowing it to differentially respond to the cell’s situation.

Whilst the pericyte contribution to neuroinflammation is being increasingly studied, little is understood regarding inflammation-related gene transcription in these cells and to date the role of C/EBPδ has not been studied in human brain pericytes. We therefore sought to investigate the function of C/EBPδ in human brain pericyte mediated inflammatory responses.

## Results

### Characterisation of adult human brain pericyte cultures

**Figure 1:**
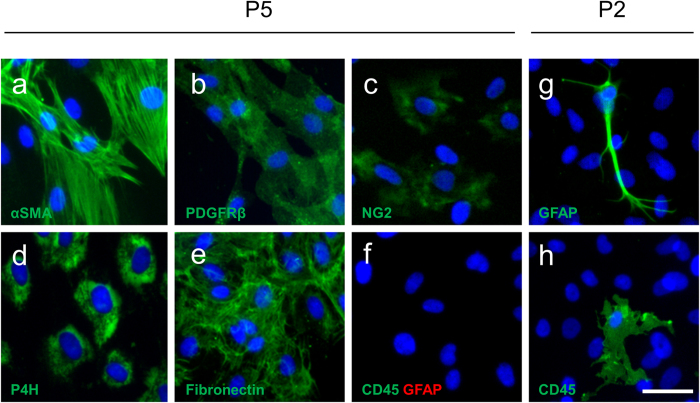
Characterisation of adult human brain pericyte cultures. Primary human brain cell cultures at passage five were stained for Hoechst (blue) and cell specific markers αSMA (**a**), PDGFRβ (**b**), NG2 (**c**), P4H (**d**), Fibronectin (**e**), CD45 and GFAP (**f**). Positive controls of astrocytes (GFAP; **g**) and microglia (CD45; **h**) at passage two are included. Scale bar = 50 μm.

Immunocytochemical analysis of early passage cell cultures obtained from human middle temporal gyrus tissue reveals a mixed population of astrocytes, microglia and pericytes as described previously^10^. Under our *in vitro* conditions microglia and astrocytes do not proliferate and are diluted out in subsequent passages. To ensure no contamination from brain glia in pericyte cultures these were grown until passage five before use. Late passage cultures showed positive immunocytochemical staining for the pericyte markers alpha smooth muscle actin (αSMA; Fig. 1a), platelet derived growth factor receptor beta (PDGFRβ; Fig. 1b) and neural/glial antigen 2(NG2; Fig. 1c) as well as the fibroblast markers prolyl-4-hydroxylase (P4H; Fig. 1d) and fibronectin (Fig. 1e). There were no cells positive for the microglia marker CD45 or the astrocyte marker glial fibrillary acidic protein (GFAP; Fig. 1f) at passage five onwards. Positive controls of GFAP (Fig. 1g) and CD45 (Fig. 1h) staining at passage two are shown.

### C/EBPδ is induced in human brain pericytes by IL-1β/IFNγ

**Figure 2:**
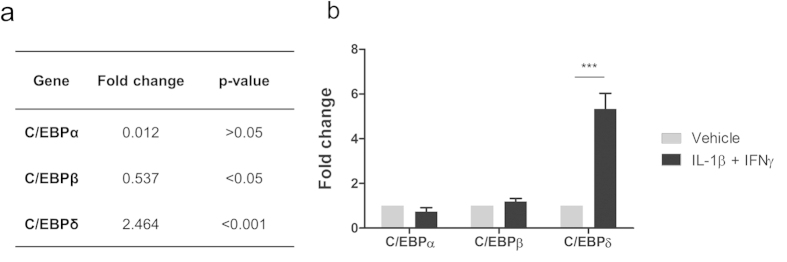
C/EBPδ is induced in human brain pericytes by IL-1β/IFNγ. Human brain pericytes were treated with vehicle or 10 ng/mL IL-1β + IFNγ for 24 hours and RNA was extracted. Expression of C/EBPα, C/EBPβ and C/EBPδ was determined by microarray^10^ (**a**) and qRT-PCR (**b**). Data is displayed as average fold change of five independent cases (a) or mean ± SEM of a separate three independent cases (**b**). ^***^ = p < 0.001.

Microarray analyis of human brain pericytes treated with IL-1β/IFNγ has previously revealed an induction of numerous inflammatory genes^10^. Due to the ability of the C/EBP family of transcription factors to modify cellular inflammatory responses, we chose to investigate the induction of three members C/EBPα, C/EBPβ and C/EBPδ in this dataset. By microarray analysis, C/EBPδ was found to be significantly increased by an IL-1β/IFNγ treatment (p < 0.001) whilst C/EBPα (p > 0.05) and C/EBPβ (p < 0.05) were not affected (Fig. 2a). Real time quantitative reverse transcriptase polymerase chain reaction (qRT-PCR) was performed on independent RNA samples, which confirmed the induction of C/EBPδ (5.33 ± 0.69 fold; p < 0.001), whereas no significant change in C/EBPα (0.74 ± 0.18 fold; p > 0.05) or C/EBPβ (1.18 ± 0.15 fold; p > 0.05) was observed (Fig. 2b), consistent with the microarray data.

### C/EBPδ is differentially induced by IL-1β, IFNγ and LPS

**Figure 3:**
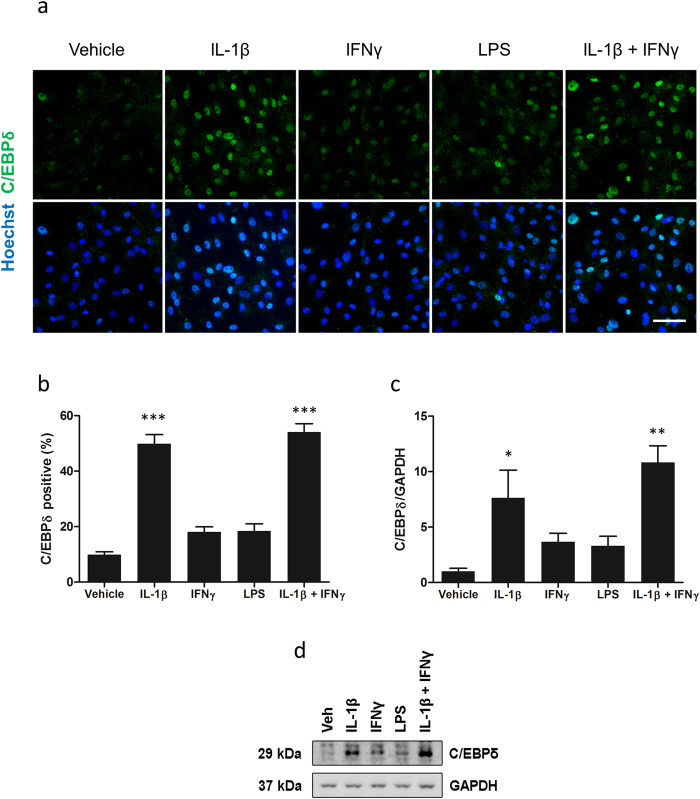
C/EBPδ is differentially induced by IFNγ, IL-1β and LPS. Human brain pericytes were treated with vehicle or 10 ng/mL IL-1β, IFNγ, or LPS for 24 hours. Representative immunocytochemistry images of C/EBPδ with treatments are shown (**a**). The percentage of cells positive for nuclear C/EBPδ was determined by immunocytochemistry (**b**) and the intensity of C/EBPδ expression was analysed by western blotting (**c,d**). Blots are cropped to improve clarity. Full-length blots are presented in Supplementary Figure S2. Data is displayed as mean ± SEM of three independent experiments. ^*^ = p < 0.05 compared to vehicle control, ^**^ = p < 0.01 compared to vehicle control ^***^ = p < 0.001 compared to vehicle control. Scale bar = 100 μm.

Having observed an induction of C/EBPδ with a combination of IL-1β and IFNγ we sought to investigate how individual inflammatory stimuli affect this response. IL-1β, IFNγ and LPS were investigated based on prior evidence for their involvement in pericyte inflammatory responses. As determined by immunocytochemistry the basal expression of C/EBPδ in pericyte cultures is low (9.85 ± 1.07%; Fig. 3a,b). Enhanced nuclear expression was observed with IL-1β alone (49.85 ± 3.36%; p < 0.001) and a combination of IL-1β and IFNγ (54.15 ± 2.96%; p < 0.001). Neither IFNγ (18.14 ± 1.80%; p > 0.05) or LPS (18.46 ± 2.57%; p > 0.05) were sufficient to significantly induce C/EBPδ expression (Fig. 3a,b). Western blot analysis revealed a similar trend with both IL-1β alone (7.63 ± 2.52 fold; p < 0.05) and in combination with IFNγ (10.82 ± 1.50 fold; p < 0.01) significantly enhancing C/EBPδ expression whilst IFNγ (3.67 ± 0.77 fold; p > 0.05) and LPS (3.32 ± 0.86 fold; p > 0.05) did not (Fig. 3c,d).

### Time course and concentration-dependant induction of C/EBPδ expression

**Figure 4:**
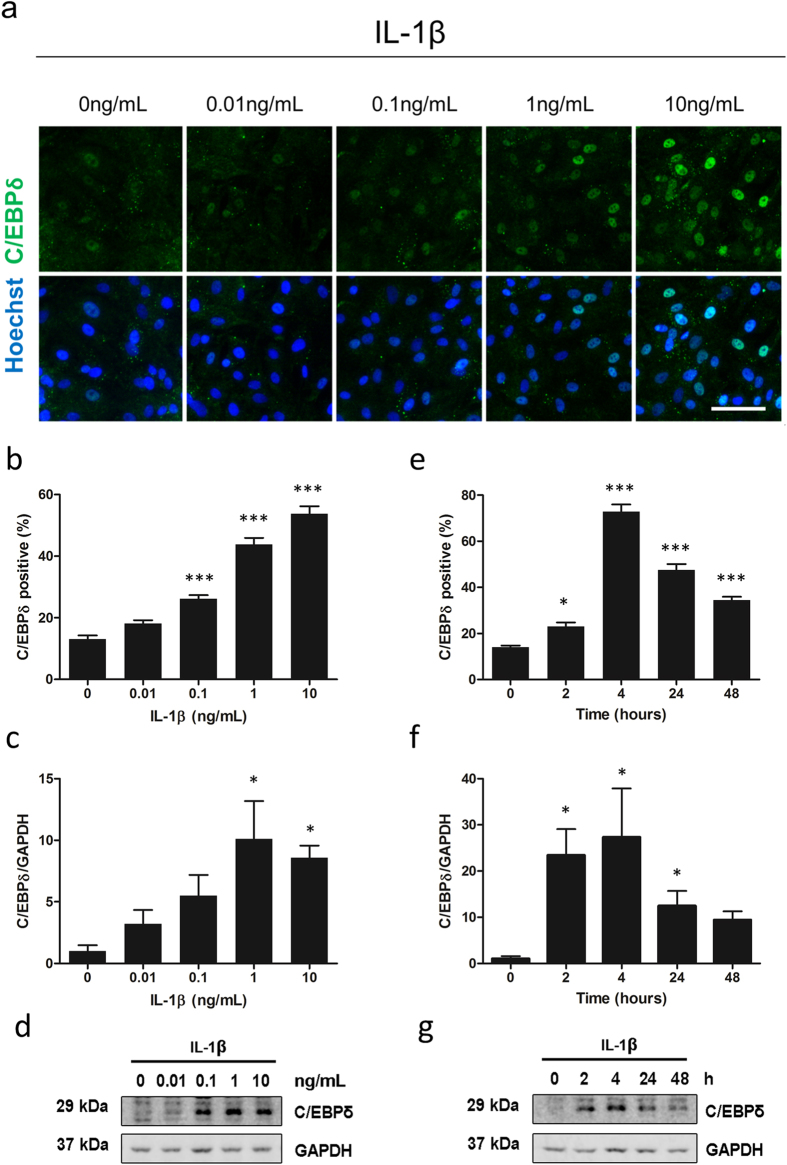
Time-course and concentration-dependant induction of C/EBPδ expression. Human brain pericytes were treated with vehicle or 0.01–10 ng/mL IL-1β for 24 hours. Representative immunocytochemistry images of C/EBPδ are shown (**a**). The percentage of cells positive for nuclear C/EBPδ was determined by immunocytochemistry (**b**) and C/EBPδ intensity was analysed by western blotting (**c,d**). Blots are cropped to improve clarity. Full-length blots are presented in Supplementary Figure S2. Human brain pericytes were treated with 10 ng/mL IL-1β for 0–48 hours. The percentage of C/EBPδ positive cells was determined by immunocytochemistry (**e**) and C/EBPδ intensity was analysed by western blotting (**f,g**). Data is displayed as mean ± SEM from three independent experiments. ^*^ = p < 0.05 compared to vehicle control, ^**^ = p < 0.01 compared to vehicle control, ^***^ = p < 0.001 compared to vehicle control. Scale bar = 100 μm.

As IL-1β treatment resulted in the greatest induction of C/EBPδ all subsequent experiments were performed using this inflammatory cytokine. To further understand the profile of C/EBPδ expression in human brain pericytes, a concentration-response curve of IL-1β induced expression was performed. Using immunocytochemistry, C/EBPδ was found to be induced by IL-1β in a concentration-dependant manner (Fig. 4a,b). A significant induction was observed by concentrations as low as 0.1 ng/mL IL-1β (vehicle 13.07 ± 1.20%, IL-1β 26.18 ± 1.16%; p < 0.001) with maximal induction by 10 ng/mL (53.73 ± 2.49%; p < 0.001). Western blotting revealed a significant induction of C/EBPδ using 1 ng/mL (10.12 ± 3.06 fold; p < 0.05) and 10 ng/mL (8.57 ± 0.99 fold; p < 0.05) IL-1β (Fig. 4c,d). In order to investigate the temporal profile of C/EBPδ induction a time-course was performed with 10 ng/mL IL-1β. By immunocytochemistry C/EBPδ was induced as early as two hours after IL-1β stimulation (vehicle 13.95 ± 0.85%, IL-1β 23.03 ± 1.71%; p < 0.05), maximally induced four hours after treatment (72.83 ± 3.11%; p < 0.001) and remained elevated 48 hours later (34.43% ± 1.45%; p < 0.001; Fig. 4e). Western blotting analysis revealed a similar trend with significant induction of C/EBPδ at two (23.42 ± 5.67 fold; p < 0.05), four (27.32 ± 10.55 fold; p < 0.05) and 24 hour treatments (12.45 ± 3.24 fold; p < 0.05) with IL-1β. However, it was not significantly elevated at 48 hours (9.43 + 1.85 fold; p > 0.05; Fig. 4f,g).

### Knockdown of C/EBPδ using siRNA

**Figure 5:**
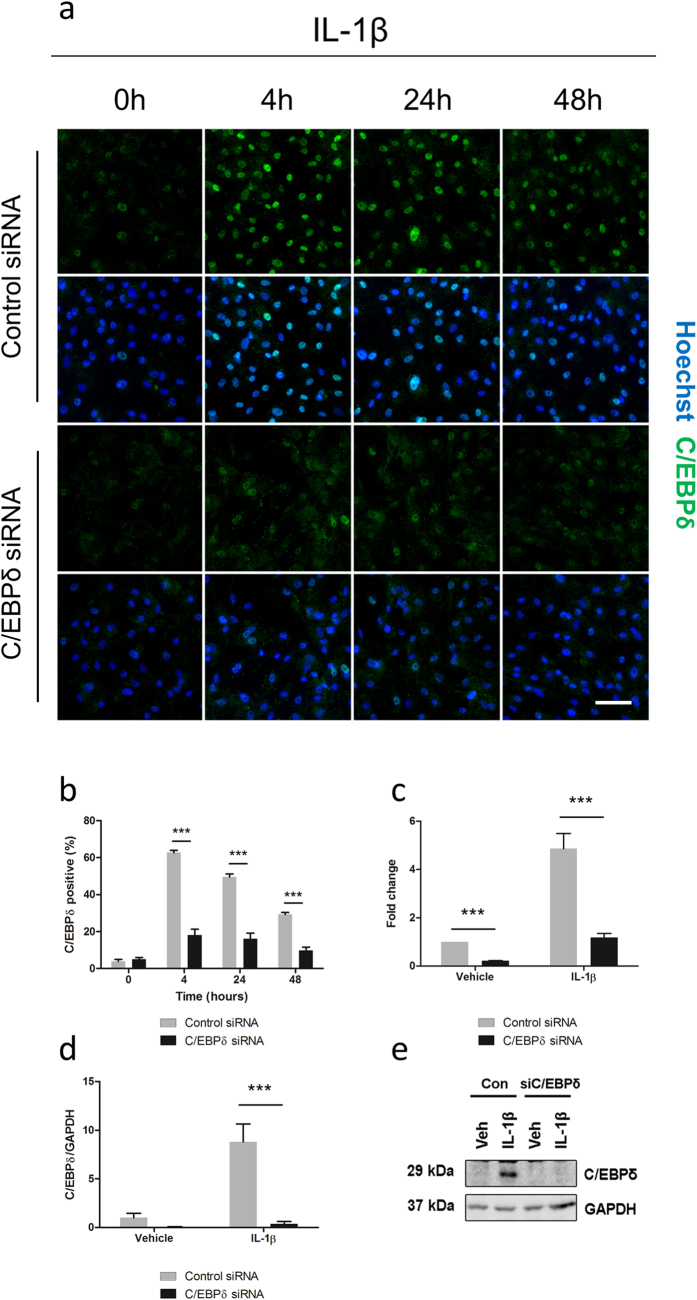
Knockdown of C/EBPδ using siRNA. Human brain pericytes were transfected with 50 nM of control or C/EBPδ siRNA for 48 hours. Following transfection, cells were treated with vehicle or 10 ng/mL IL-1β for 4–48 hours. Representative immunocytochemistry images of C/EBPδ with treatments are shown (**a**) and the percentage of cells positive for nuclear C/EBPδ was quantified (**b**). RNA was extracted following a six hour treatment with IL-1β and C/EBPδ transcript expression was determined via RT-qPCR (**c**). Protein was extracted following a 24 hour treatment with IL-1β and C/EBPδ expression determined by western blotting (**d,e**). Blots are cropped to improve clarity. Full-length blots are presented in Supplementary Figure S2. ^***^ = p < 0.001. Scale bar = 100 μm.

C/EBPδ has been widely reported to modify inflammatory gene expression in various cell types. To investigate its effects on human brain pericyte inflammatory responses, a C/EBPδ siRNA construct was employed. Transfection of 50 nM C/EBPδ siRNA significantly reduced IL-1β induced C/EBPδ expression at four hours (control siRNA 62.70 ± 1.18%, C/EBPδ siRNA 18.17 ± 3.07%; p < 0.001), 24 hours (control siRNA 49.63 ± 1.51%, C/EBPδ siRNA 16.14 ± 3.05%; p < 0.001) and 48 hours (control siRNA 29.26 ± 1.01%, C/EBPδ siRNA 9.71 ± 1.89%; p < 0.001), however, had no effect on basal levels (control siRNA 3.89 ± 0.98%, C/EBPδ siRNA 4.99 ± 0.99%; p > 0.05) as determined by immunocytochemistry (Fig. 5a,b). Using qRT-PCR a reduction in both basal (0.22 ± 0.02 fold; p < 0.001) and IL-1β induced gene expression (control siRNA 4.86 ± 0.62 fold, C/EBPδ siRNA 1.18 ± 0.17; p < 0.001; Fig. 5c) was observed with C/EBPδ siRNA. Western blotting analysis revealed no basal change in C/EBPδ with siRNA treatment (0.05 ± 0.03 fold; p > 0.05), however a significant attenuation of IL-1β induced expression was observed (control siRNA 8.80 ± 1.85 fold, C/EBPδ siRNA 0.37 ± 0.23; p < 0.001; Fig. 5d,e).

### C/EBPδ knockdown modifies IL-1β induced inflammatory gene expression

**Figure 6:**
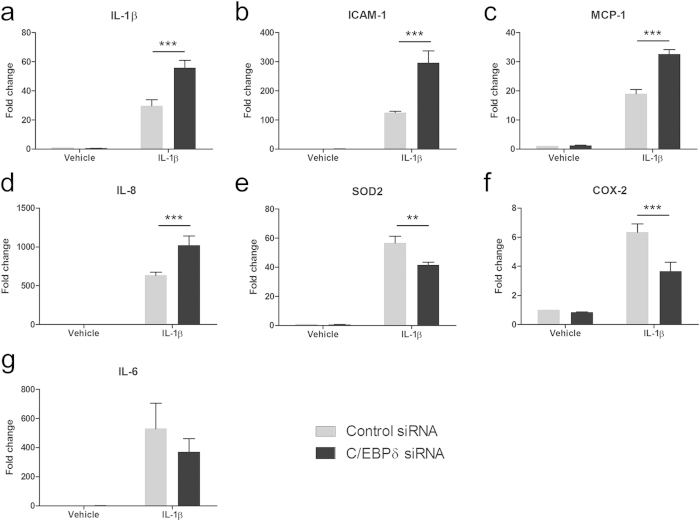
C/EBPδ knockdown modifies IL-1β induced inflammatory gene expression. Human brain pericytes were transfected with 50 nM of control or C/EBPδ siRNA for 48 hours. Following transfection, cells were treated with vehicle or 10 ng/mL IL-1β for six hours and RNA was extracted. The effect of C/EBPδ knockdown on IL-1β (a), ICAM-1 (**b**), MCP-1 (**c**), IL-8(**d**), SOD2 (**e**), COX-2 (**f**) and IL-6 (**g**) was assayed by qRT-PCR. Data is displayed as mean ± SEM from four independent cases ^**^ = p < 0.01, ^***^ = p < 0.001.

Having observed a reduction in IL-1β induced C/EBPδ expression with siRNA transfection, we examined the effect on a range of inflammatory mediators which have previously been shown to be induced by brain pericytes with inflammatory stimuli. C/EBPδ knockdown resulted in increased IL-1β-induced expression of IL-1β (control siRNA 29.74 ± 4.13 fold, C/EBPδ siRNA 55.88 ± 4.99 fold; p < 0.001; Fig. 6a), ICAM-1 (control siRNA 125.20 ± 5.40 fold, C/EBPδ siRNA 295.72 ± 41.51 fold; p < 0.001; Fig. 6b), MCP-1 (control siRNA 19.04 ± 1.39 fold, C/EBPδ siRNA 32.59 ± 1.61 fold; p < 0.001; Fig. 6c) and IL-8 (control siRNA 634.81 ± 38.01 fold, C/EBPδ siRNA 1021.12 ± 121.10 fold; p<0.001; Fig. 6d). In contrast, attenuated expression was observed for SOD2 (control siRNA 56.72 ± 4.47 fold, C/EBPδ siRNA 41.50 ± 1.98 fold; p < 0.01; Fig. 6e) and COX-2 (control siRNA 6.35 ± 4.86 fold, C/EBPδ siRNA 4.50 ± 1.14 fold; p < 0.001; Fig. 6f), whilst a non-significant decrease was observed for IL-6 (control siRNA 530.91 ± 174.61 fold, C/EBPδ siRNA 369.92 ± 133.60 fold; p > 0.05; Fig. 6g). The basal expression of all inflammatory genes showed no change with C/EBPδ or control siRNA.

### C/EBPδ knockdown enhances IL-1β induced ICAM-1 and MCP-1 expression

**Figure 7:**
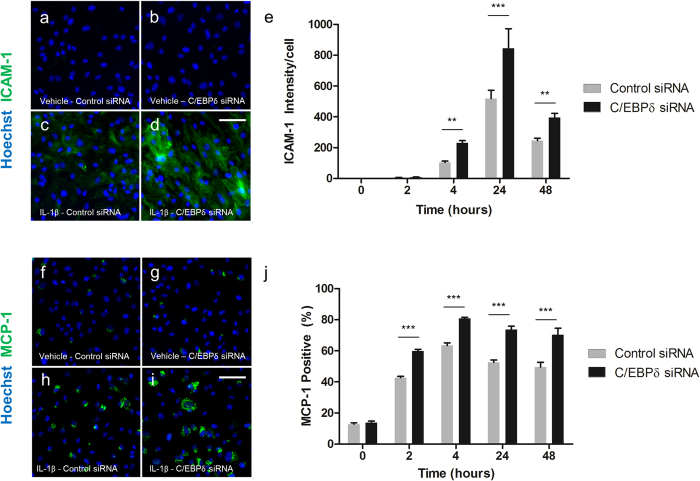
C/EBPδ knockdown enhances IL-1β induced MCP-1 and ICAM-1 protein expression. Human brain pericytes were transfected with 50 nM of control or C/EBPδ siRNA for 48 hours. Following transfection, cells were treated with vehicle or 10 ng/mL IL-1β for 2–24 hours and cells fixed and immunostained for ICAM-1 and MCP-1. Representative images of ICAM-1 and MCP-1 immunostaining respectively with vehicle and control siRNA (**a,f**), vehicle with C/EBPδ siRNA (**b,g**), 24 hours IL-1β with control siRNA (**c,h**) and 24 hours IL-1β with C/EBPδ siRNA are shown (**d,i**). Intensity of ICAM-1 staining (**e**) and the percentage of MCP-1 positive cells (j) were determined. Data is displayed as mean ± SEM from three independent experiments. ^**^ = p < 0.01, ^***^ = p < 0.001. Scale bar = 100 μm.

In order to determine whether changes at the RNA level correlated with altered protein expression, immunocytochemistry for ICAM-1 and MCP-1 was performed. Unstimulated pericytes showed low basal expression of ICAM-1 and this was unaffected by C/EBPδ siRNA (1.00 ± 0.10 AU and 1.82 ± 0.30 AU respectively; Fig. 7a,b,e). A significant induction of ICAM-1 expression was observed with IL-1β treatment at four (104.61 ± 7.98AU; p < 0.001; Fig. 7e), 24 (518.92 ± 53.80AU; p < 0.001; Fig. 7c,e) and 48 hours (245.31 ± 15.61AU; p < 0.001; Fig. 7e). Compared to the control siRNA, C/EBPδ siRNA further increased IL-1β stimulated ICAM-1 expression at four (230.93 ± 14.85AU; p < 0.01; Fig. 7e), 24 (843.56 ± 128.70AU; p < 0.001; Fig. 7c, e) and 48 hours (394.52 ± 27.55AU; p < 0.01; Fig. 7e). Immunocytochemical analysis of MCP-1 demonstrated low basal levels which were also unaffected by C/EBPδ siRNA (12.77 ± 0.89% and 13.77 ± 1.03% respectively; Fig. 7f,g,j). The percentage of MCP-1 positive cells was significantly increased following IL-1β treatment at two (42.73 ± 0.81%; p < 0.001; Fig. 7j), four (63.47 ± 1.62%; Fig. 7j), 24 (52.51 ± 1.61%; Fig. 7h,j) and 48 hours(49.56 ± 2.97%; Fig. 7j). Compared to control siRNA, C/EBPδ siRNA further increased IL-1β stimulated MCP-1 expression at each of the measured time point (2 hours, 59.83 ± 1.13%; four hours 80.67 ± 0.83%; 24 hours 73.51 ± 2.25%; 48 hours 70.39 ± 4.15%; p < 0.001; Fig. 7i,j).

### C/EBPδ knockdown modifies pericyte secretion of inflammatory mediators

**Figure 8:**
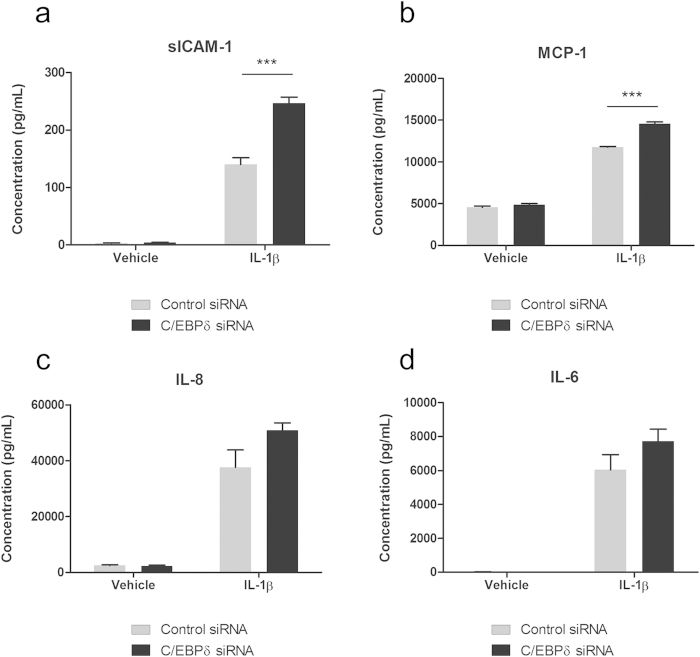
C/EBPδ knockdown modifies pericyte secretion of inflammatory mediators. Human brain pericytes were transfected with 50 nM of control or C/EBPδ siRNA for 48 hours. Following transfection cells were treated with vehicle or 10 ng/mL IL-1β for 24 hours and conditioned media collected. Concentration of sICAM-1 (**a**), MCP-1 (**b**), IL-8 (**c**) and IL-6 (**d**) in media was determined using a multiplex cytometric bead array. Data represent mean ± SEM (n = 4). ^***^ ^=^ p < 0.001.

Secreted cytokines and chemokines are essential in modulating cellular cross-talk and inflammatory responses. In order to determine whether the observed changed in mRNA and protein expression correlated with increased secretion, cytokine concentrations in the pericytes conditioned media were measured using a cytometric bead array (CBA). Unstimulated pericytes demonstrated basal expression of soluble ICAM-1 (sICAM-1; 3.13 ± 0.75 pg/mL), MCP-1 (4,560.79 ± 143.38 pg/mL) , IL-8 (2,483.51 ± 202.58 pg/mL) and IL-6 (22.75 ± 2.80 pg/mL), which were not significantly altered by C/EBPδ siRNA (ICAM-1 4.15 ± 0.3 pg/mL; p > 0.05, MCP-1 4,837.74 ± 188.27 pg/mL ; p > 0.05, IL-8 2,345.53 ± 206.12 pg/mL ; p > 0.05 and IL-6 21.25 ± 2.66 pg/mL; p > 0.05; Fig. 8a–d). IL-1β treatment significantly increased the concentration of all measured cytokines in the media (ICAM-1 139.90 ± 11.85 pg/mL; p < 0.001, MCP-1 11,755.35 ± 69.58 pg/mL; p < 0.001, IL-8 37,616.57 ± 10,815.63 pg/mL; p < 0.001 and IL-6 6,040.13 ± 885.67 pg/mL; p < 0.001; Fig. 8a–d). Compared to the control siRNA condition, C/EBPδ siRNA significantly enhanced the IL-1β induced secretion of ICAM-1 (246.5 ± 10.93 pg/mL; p < 0.001; Fig. 8a) and MCP-1 (14,551.65 ± 205.73 pg/mL; p < 0.001; Fig. 8b); however, did not alter IL-8 (50,833.75 ± 2,719.64 pg/mL; p > 0.05; Fig. 8c) or IL-6 secretion (7,724.97 ± 705.35 pg/mL; p > 0.05; Fig. 8d).

## Discussion

C/EBPδ was found to be induced in human brain pericyte cultures by the pro-inflammatory cytokine IL-1β. Several studies have reported induction of C/EBPδ expression in microglia and astrocytes following inflammation^25^,^26^,^29^, however, C/EBPδ expression in pericytes has not been previously shown. Using C/EBPδ specific siRNA we were able to attenuate the induction of pericyte C/EBPδ following immune challenge in order to identify its contribution to the inflammatory response.

Consistent with literature from rodent microglia and astrocytes, C/EBPδ knock-down attenuated COX-2 expression; however it did not significantly alter IL-6 expression^25^,^26^. COX-2 is an inducible enzyme that catalyses prostaglandin formation, many of which have roles in inflammation. Whilst typically thought to have a pro-inflammatory response, anti-inflammatory roles of prostaglandins are now being acknowledged^36^. Prostaglandin I2 (PGI2) is the most prominent prostaglandin synthesised by cells of the vasculature including endothelial cells and vascular smooth muscle cells and is predominantly synthesised via COX-2^36^,^38^. Furthermore, PGI2 is the major prostaglandin product from retinal pericyte cells^39^. PGI2 inhibits leukocyte adhesion to vascular endothelium and therefore may attenuate immune infiltration^40^. In order to understand the specific inflammatory role of COX-2 induction in pericytes the composition of prostaglandins needs to be studied in context of the system. Similarly, a dual role in both anti-inflammatory and pro-inflammatory responses has been observed for IL-6 with evidence supporting a neuroprotective role in the brain^41^,^42^. Care should be taken when classifying these as either pro or anti-inflammatory due to their context dependant nature.

Attenuation of IL-1β induced SOD-2 expression was also observed with C/EBPδ knock-down. Induction of SOD-2 by a range of inflammatory factors has previously been shown, whilst its promoter region contains a C/EBP binding site^43^,^44^. SOD-2 regulates oxidative stress in cells by converting superoxide into hydrogen peroxide. Superoxide ions enhance recruitment of immune cells, precipitate DNA damage and were found to augment behavioural deficits in a mouse model of Alzheimer’s disease^45^,^46^,^47^. Furthermore, oxidative stress is believed to be a major contributor to neuronal damage in inflammatory conditions and elimination of superoxide formation by SOD-2 is often considered to be anti-inflammatory^48^. The finding that C/EBPδ enhances SOD-2 induction may indicate an anti-oxidant/anti-inflammatory role for this transcription factor following inflammation.

Interestingly, C/EBPδ knock-down was also found to enhance the expression of several IL-1β induced inflammatory genes including IL-8, ICAM-1, MCP-1 and IL-1β. Two of which, MCP-1 and ICAM-1, were confirmed to be upregulated by immunocytochemical staining, as well as secretions into culture media. MCP-1 is a major chemokine involved in the recruitment of monocytes. In the context of CNS immune responses, secretions of MCP-1 from brain pericytes could be involved in the trafficking of monocytes from blood into the brain.^49^,^50^. Several parenchymal brain cells produce MCP-1 under inflammatory conditions, including microglia, astrocytes and pericytes^10^. Peripheral immune cell recruitment to the brain may further enhance central inflammatory responses through potentiated inflammatory cytokine production. Furthermore, MCP-1 increases both local microglial proliferation, as well as migration to injured sites^19^. As the brain’s predominant immune cell, increased microglial presence and activation may further worsen inflammatory responses, contributing to neuronal death. Indeed, enhanced MCP-1 expression has been observed in several neurological disorders and may worsen disease progression^51^,^52^,^53^. Following inflammatory responses, the finding that C/EBPδ induction acts to dampen MCP-1 expression in brain pericytes suggests potential for reducing peripheral immune cell infiltration. Interestingly, the only study currently proposing an anti-inflammatory role of C/EBPδ utilised pancreatic beta cells to show that C/EBPδ knock-down enhanced IL-β/IFNγ induced chemokine expression, corroborating the changes we see in MCP-1 expression^33^. Investigations into the effect of C/EBPδ on brain glia chemokine expression are currently lacking.

ICAM-1 is a transmembrane protein involved in stabilising cell-cell interactions^15^. It is a ligand for the CD11 family of leukocyte adhesion molecules, which are widely expressed and inducible on leukocytes and this interaction aids their attachment to brain vasculature and subsequent migration into tissue^54^. Under basal conditions ICAM-1 expression in unstimulated pericytes is low; however it was found to be significantly elevated by the pro-inflammatory cytokine IL-1β. Expression of ICAM-1 on pericytes has previously been shown to aid peripheral immune cell infiltration across tissue vasculature, potentially worsening CNS inflammatory responses^8^,^16^,^54^. The finding that C/EBPδ knockdown enhances ICAM-1 expression on brain pericytes suggests that C/EBPδ induction during immune challenge may be beneficial in preventing ICAM-1mediated immune cell infiltration, highlighting another anti-inflammatory role for this transcription factor. Interestingly, C/EBPβ knockdown in human astrocytes was also found to enhance IL-1β induced ICAM-1 expression suggesting that this protein is largely regulated by C/EBP family members^24^.

C/EBPδ knockdown also enhanced IL-1β induced IL-8 and IL-1β gene expression. IL-8 is a potent neutrophil chemokine that has previously been observed in brain pericytes and involved in enhanced CNS immune cell infiltration^8^,^55^. Like MCP-1 and ICAM-1, it represents another mechanism whereby pericytes can enhance central infiltration of peripheral immune cells. Interestingly, the change in IL-8 mRNA expression with C/EBPδ knockdown did not translate to a significant change in protein secretion. The reasons for this are currently unclear. IL-8 was the most concentrated of all secreted proteins measured in culture media and perhaps saturation of secretory pathways was observed at the measured time-point. C/EBPδ regulation of IL-8 therefore warrants further investigation.

Due to the use of IL-1β to stimulate pericytes, its expression in culture media was unable to be measured. IL-1β is a potent inflammatory stimulus, and therefore its induction following C/EBPδ knockdown would appear to contradict several of the anti-inflammatory roles in pericytes. However, pericyte stimulation with other inflammatory stimuli (IFNγ, LPS, TNFα and IL-6) revealed an increase in IL-1β mRNA, which did not translate to IL-1β secretion (Fig. S1). It is possible then that the observed IL-1β gene change with C/EBPδ knockdown would not necessarily cause increased IL-1β secretion. Indeed no evidence for pericyte secretion of IL-1β exists from studies examining their secretome^7^,^8^.

In conclusion, we propose that C/EBPδ has a role in modifying inflammatory responses of human brain pericytes. Contrary to previous data involving brain glia, we have demonstrated an anti-inflammatory role for C/EBPδ induction in the CNS. This is achieved through limiting pericyte MCP-1 and ICAM-1 expression, which in turn may attenuate peripheral immune cell infiltration into the CNS. Of course whether these changes induced by C/EBPδ knock-down would actually lead to altered inflammatory events *in vivo* is not clear as currently we don’t know whether the concentrations of cytokines/chemokines measured *in vitro* are biologically relevant. Despite this, the involvement of pericytes in neuroinflammation is becoming increasingly recognised and further studies investigating opportunities to control this response are warranted.

## Methods

### Tissue source

Human middle temporal gyrus brain tissue was obtained, with informed consent, from surgeries of patients with drug-resistant temporal lobe epilepsy. All specimens were collected with written patient consent. All protocols used in this study were approved by the Northern Regional Ethics Committee (New Zealand) and all methods were carried out in accordance with the approved guidelines.

### Mixed glial cultures isolated from human brain tissue

Mixed glial cultures containing microglia, astrocytes and pericytes were isolated from adult human brain tissue as per^56^ with minor modifications. Approximately 2 g of tissue from the middle temporal gyrus was washed in Hanks balanced salt solution (HBSS; Ca^2+^ and Mg^2+^ free, Gibco BRL, CA, USA), meninges and visible blood vessels removed and tissue diced into pieces approximately 1 mm^3^. Tissue was added to 10 mL g^−1^ enzyme dissociation mix (10 U mL^−1^ DNase (Invitrogen, CA, USA) and 2.5 U mL^−1^ papain (Worthington, NJ, USA) in Hibernate-A medium (Gibco, CA, USA) for 15 minutes at 37 °C with gentle rotation, triturated to aid digestion and incubated for a further 15 minutes. The tissue was further triturated, allowed to settle and the supernatant transferred to a new tube containing equal volume Dulbecco’s modified Eagle medium: Nutrient mixture F-12 (DMEM/F12) (Gibco, CA, USA) supplemented with 10% foetal bovine serum (FBS; Gibco, CA, USA) and 1% Penicillin-Streptomycin-Glutamine (PSG; Gibco, CA, USA) henceforth referred to as DMEM/F12. The cell suspension was passed through a 100 μm nylon cell strainer (Becton Dickenson, NJ, USA), centrifuged at 1000 RPM for 10 minutes and the pellet resuspended in DMEM/F12. 20 mL of the single cell suspension was added to T75 tissue culture flasks (Nunc, Roskilde, Denmark) and incubated overnight at 37 °C with 95% air / 5%CO_2_. The following day, media from flask containing unattached cells and debris was removed and centrifuged at 1000 RPM for 10 minutes. The resulting pellet was resuspended in DMEM/F12 and added back to the flask. On day three, media containing unattached cells or debris was removed and discarded. Cells were grown until confluent (1–2 weeks) at which point they were harvested with 0.25% Trypsin-1mM Ethylenediaminetetraacetic acid (EDTA) (Gibco, CA, USA) and gentle scraping. Cells were passaged up to passage eight with early passages (two-three) containing microglia, astrocytes and pericytes, whilst latter passages (four-eight) contained only pericytes. Detailed characterisation of both early and late passage cultures has been performed previously^10^. Briefly, late passage cultures were positive for pericyte markers α-smooth muscle actin, platelet derived growth factor receptor-β and neural/glial antigen-2 and showed no expression of the astroglial marker glial fibrillary acidic protein (GFAP) or microglial markers PU.1 and CD45. However, although we have used a number of markers, none of these are specific to pericytes so it is possible that our cultures contain other fibroblast-like cells. For simplicity, however, we will refer to late passage cultures as pericytes. We have also quantified the relative proportions of microglia, astrocytes, and pericytes in our cultures at different passages. In general (there is some variation between different donors), we find ~10% CD45-positive microglia, ~5% GFAP-positive astrocytes, and 85% pericytes in passage two cultures. Passage five and above cultures contain 100% pericytes and thus were used for all experiments.

### Drug and immunogen treatments

To induce inflammatory responses, cells were treated with indicated concentrations (10 pg/mL-10 ng/mL) of IFNγ (R&D Systems, MN, USA), IL-1β (Peprotech, NJ, USA), LPS (from *Escherichia coli* 026:B6, L4391, Sigma, MO, USA) or vehicle (0.1% BSA in PBS) for indicated times (0–48 hours).

### siRNA transfection

Synthetic C/EBPδ siRNA (Santa Cruz, sc-37722, CA, USA), or a control siRNA (Santa Cruz, sc-37007) was incubated with Lipofectamine® RNAiMAX (Life Technologies, CA, USA) in DMEM/F12 with 10% FBS for 20 minutes at room temperature. 50 nM siRNA was added to cells for 48 hours.

### Collection of conditioned media and cytokine measurement by cytometric bead array

Conditioned media was collected from cells grown in a 96 well plate. Media was spun at 16 x g for five minutes to collect possible cells and debris. Supernatant was obtained and stored –20°C. The concentration of cytokines was measured using cytometric bead array (CBA)(BD Biosciences, CA, USA) as described previously^57^. CBA samples were run on an Accuri C6 flow-cytometer (BD Biosciences, CA, USA). Data was analysed using FCAP-array software (version 3.1) (BD Biosciences, CA, USA) to convert fluorescent intensity values to concentrations.

### Immunocytochemistry

Cells were fixed in 4% paraformaldehyde for 15 minutes and washed in phosphate buffered saline with 0.1% triton X-100 (PBS-T). Cells were incubated with primary antibodies (Table S1) overnight at 4 °C in immunobuffer containing 1% goat serum, 0.2% triton X-100 and 0.04% thimerosal in PBS. Cells were washed in PBS-T and incubated with appropriate anti-species fluorescently conjugated secondary antibodies overnight at 4 °C. Cells were washed again and incubated with Hoechst stain for 20 minutes. Images were acquired using the Discovery-1 automated fluorescence microscope (Molecular Devices) or ImageXpress® Micro XLS (Version 5.3.0.1, Molecular Devices, CA, USA). Quantitative analysis of intensity measures and positively stained cells was performed using the cell scoring and integrated morphometry analysis modules on Metamorph® software (Version 6.2.6, Molecular Devices, CA, USA) for Discovery-1 acquired images and MetaXpress® software (Version 5.3.0.1, Molecular Devices, CA, USA) for Image Xpress® Micro XLS acquired images. Roughly 500–1000 cells were scored per well with multiple wells (at least four) analysed per sample.

### Western blotting and densitometry analysis

Following treatments, cells were harvested by rinsing in ice-cold PBS, scraping directly into sample harvesting buffer (Laemmli buffer^58^ without bromophenol blue or reducing agents) and boiling for 10 minutes. Protein concentrations were quantified using the BioRad DC Protein Assay (BioRad, Hercules, CA, USA). Samples were diluted to give 20 or 30 μg protein per lane in sample harvesting buffer with final 100 mM DTT and 0.02% bromophenol blue and boiled for a further 10 minutes. Samples were then resolved by electrophoresis on 4–12% NuPAGE Bis-Tris SDS-PAGE gels (Life Technologies). Gels were transferred onto Immobilon-FL PVDF membrane (Millipore) using the X-Cell II blot module (Life Technologies). Blots were blocked in 5% non-fat dried milk in Tris-buffered saline with 0.1% tween (TBS-T) for 30 min. Blots were probed overnight at 4 °C with primary antibodies, then for two hours at room temperature with secondary antibodies (all in 50% Odyssey blocking buffer in TBS-T, see Table S1 for antibody details). Blots were scanned on the Li-Cor Odyssey Fc Imager (Li-Cor Biotechnology, Lincoln, NE, USA). Blot images in TIF format were quantified using the gel analyser tool in ImageJ (version 1.49d, NIH, Bethesda, USA, http://rsb.info.nih.gov/ij/). Integrated band intensities were normalised to band intensities of GAPDH loading controls.

### Real time quantitative reverse transcriptase PCR

Cells were washed in PBS and RNA extraction and purification was performed using the RNeasy® mini kit (Qiagen, Limberg Netherlands) as per manufacturer’s instructions. RNA was treated with DNase (1 μg DNase/1 μg RNA) using the RQ1 RNase-free DNAse kit (Promega, WI, USA) and cDNA made using the Superscript® First-Strand Synthesis kit (Life Technologies, CA, USA). Quantitative real-time reverse transcriptase polymerase chain reaction was performed using Platinum® SYBR® Green qPCR SuperMix-UDG with Rox (Life Technologies, CA, USA). Standard curves were run for all primers used and efficiencies were all 100 ± 10% (Table S2). Relative gene expression analysis was performed using the ΔΔC_t_ method to the housekeeping gene GAPDH.

### Microarray

Microarray experiment was performed as described in^10^. Briefly, cells destined for microarray experiment where treated for 24 hours with vehicle (0.1% BSA in PBS) or 10 ng/mL IL-1β/IFNγ. Cells were washed in PBS and RNA extracted as above for qRT-qPCR experiments. RNA quality was analysed using the Experion™ system (Bio-Rad, CA, USA) and the Experion™ RNA StdSens Analysis kit. RNA was labelled and hybridised to Affymetirx Genechip® Primeview™ Human Gene Expression Arrays (Santa Clara, CA, USA) according to manufacturer’s instructions.

### Statistical analysis

Unless otherwise stated, all experiments were performed at least three independent times from three different tissue donors. Statistical analysis was carried out using one-way analysis of variance (ANOVA) followed by Dunnett’s multiple comparison test to compare treatments versus vehicle control (Graphpad Prism 5.02). For siRNA experiments a Two-way ANOVA with Bonferroni post–test was used to compare between treatments. For statistical analysis of qPCR data ΔCt values were used. Statistical analysis of microarray experiment was performed as previously described^10^.

## Additional Information

**How to cite this article**: Rustenhoven, J. *et al.* An anti-inflammatory role for C/EBPδ in human brain pericytes. *Sci. Rep.* **5**, 12132; doi: 10.1038/srep12132 (2015).

## Supplementary Material

Supplementary Information
