# Supplementary material for: An anti-inflammatory role for C/EBPδ in human brain pericytes
Source: Sci Rep. 2015 Jul 13;5:12132. doi: 10.1038/srep12132 (PMC4499812; doi:10.1038/srep12132)
Supplement: Supplementary Information [file srep12132-s1.docx]

**An anti-inflammatory role for C/EBPδ in human brain pericytes**

Justin Rustenhoven ^1,4^, Emma L. Scotter ^1,4^, Deidre Jansson ^1, 2, 4^, Dan T Kho ^1,4^, Robyn L. Oldfield ^6^, Peter S. Bergin ^4, 7^, Edward W. Mee ^4, 7^, Richard L. M. Faull ^3, 4^, Maurice A. Curtis ^3,4^ , Scott E. Graham ^1, 4^ Thomas I-H. Park ^1, 4^, Mike Dragunow ^1, 2, 4^.

**Table S1: List of antibody dilutions**

| Antibody | Company | Catalogue # | Dilution |
| --- | --- | --- | --- |
| Mouse anti-C/EBPδ | Santa Cruz | sc-365546 | 1:250 |
| Rabbit anti-C/EBPδ | LS Bio | LS-B10190 | 1:500 |
| Mouse anti-NG2 | Santa Cruz | sc-53389 | 1:500 |
| Mouse anti-HLA,DR,DP,DQ | DAKO | M0775 | 1:500 |
| Mouse anti-COX2 | BD Pharminogen | 610204 | 1:250 |
| Mouse anti-ICAM-1 | Santa Cruz | sc-107 | 1:500 |
| Rabbit anti-αSMA | Abcam | Ab5694 | 1:100 |
| Mouse anti-CD45 | Abcam | Ab8216 | 1:500 |
| Rabbit anti-Fibronectin | DAKO | A0245 | 1:10,000 |
| Rabbit anti-PDGFRβ | Cell Signalling | mAb3169 | 1:500 |
| Rabbit anti-P4H | Sigma | HPA0075991 | 1:500 |
| Rabbit anti-GFAP | DAKO | Z0334 | 1:10,000 |
| Rabbit anti-MCP1 | Abcam | Ab74121 | 1:500 |
| Mouse anti-GAPDH | Abcam | ab9484 | 1:1,500 |
| Goat anti-mouse Alexa Fluor® 488 | Invitrogen | A11001 | 1:500 |
| Goat anti-mouse Alexa Fluor® 594 | Invitrogen | A11005 | 1:500 |
| Goat anti-rabbit Alexa Fluor® 488 | Invitrogen | A11008 | 1:500 |
| Goat anti-rabbit Alexa Fluor® 594 | Invitrogen | A11012 | 1:500 |
| Goat anti-mouse IRDye-680LT | LiCOR | 926-68020 | 1:20,000 |
| Goat anti-rabbit IRDye-800CW | LiCOR | 926-32211 | 1:20,000 |

**Table S2: List of qPCR primers used**

| Gene |  | Sequence | Amplicon Length | | Primer Efficiency | |
| --- | --- | --- | --- | --- | --- | --- |
| GAPDH (h) | Fw | CATGAGAAGTATGACAACAGCCT | | 113 bp | | 98.4% |
|  | Rv | AGTCCTTCCACGATACCAAAGT | |  | |  |
| ICAM1 (h) | Fw | GAACCAGAGCCAGGAGACAC | | 84 bp | | 100.9% |
|  | Rv | GAGACCTCTGGCTTCGTCAG | |  | |  |
| IL-6 (h) | Fw | TTCGGTCCAGTTGCCTTCTC | | 77 bp | | 98.3% |
|  | Rv | TCTTCTCCTGGGGGTACTGG | |  | |  |
| IL-8 (h) | Fw | CAGAGACAGCAGAGCACACA | | 70 bp | | 102.0% |
|  | Rv | GTGAGATGGTTCCTTCCGGT | |  | |  |
| SOD-2 (h) | Fw | GCCCTGGAACCTCACATCAA | | 79 bp | | 100.6% |
|  | Rv | TCAGGTTGTTCACGTAGGCC | |  | |  |
| COX-2 (h) | Fw | AGGGTTGCTGGTGGTAGGAA | | 76 bp | | 97.7% |
|  | Rv | TCTGCCTGCTCTGGTCAATG | |  | |  |
| C/EBPδ (h) | Fw | TTCAGCGCCTACATCGACTC | | 80 bp | | 94.9% |
|  | Rv | TTGAAGAGGTCGGCGAAGAG | |  | |  |
| C/EBPβ(h) | Fw | AGAGCAAGGCCAAGAAGAC | | 77 bp | | 93.9% |
|  | Rv | CACGGCGATGTTGTTGC | |  | |  |
| C/EBPα (h) | Fw | GCCGGGAGAACTCTAACTCC | | 82 bp | | 91.2% |
|  | Rv | TGCAGGTGGCTGCTCAT | |  | |  |
| IL-1β (h) | Fw | CTCACTTAAAGCCCGCCTGA | | 70 bp | | 94.1% |
|  | Rv | GGAGCGAATGACAGAGGGTT | |  | |  |
| MCP-1 (h) | Fw | CAGCCAGATGCAATCAATGCC | | 190 bp | | 92.8% |
|  | Rv | TGGAATCCTGAACCCACTTCT | |  | |  |

a


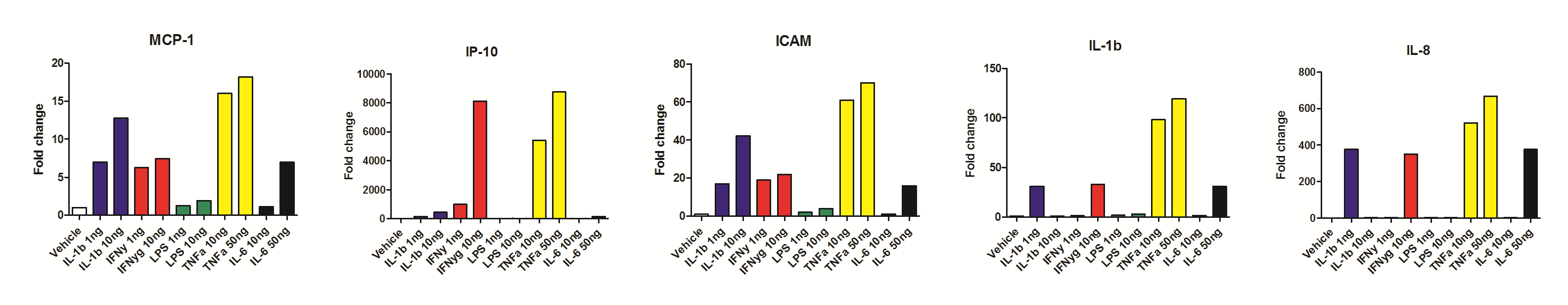

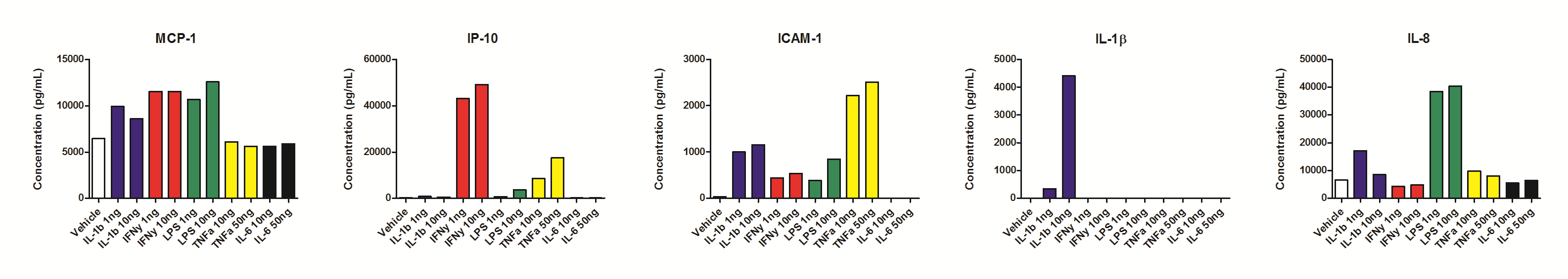

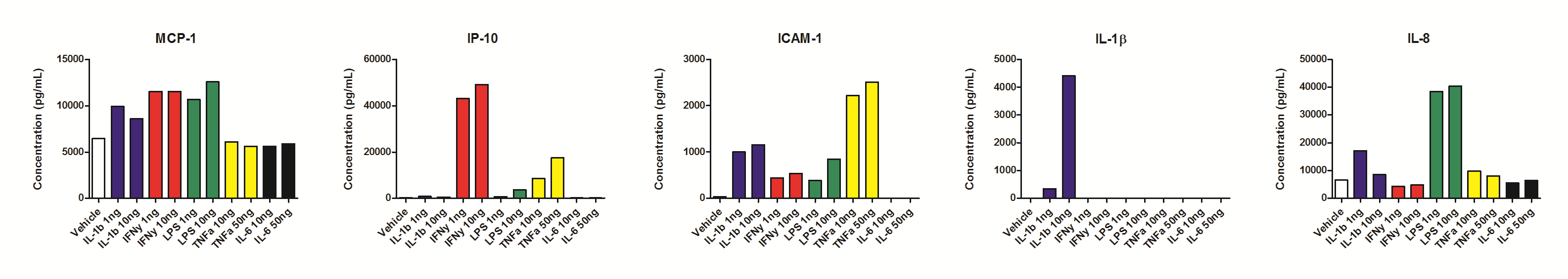


b

**Figure S1. Comparison of RNA changes with cytokine secretion following inflammatory stimulation.** Human brain pericytes were treated with a vehicle or 1-50 ng/mL of IL-1β, IFNγ, LPS TNFα or IL-6 for 24 hours and conditioned media collected and cytokine secretion determined by CBA (a). From the same samples RNA was extracted and qRT-PCR performed (b).


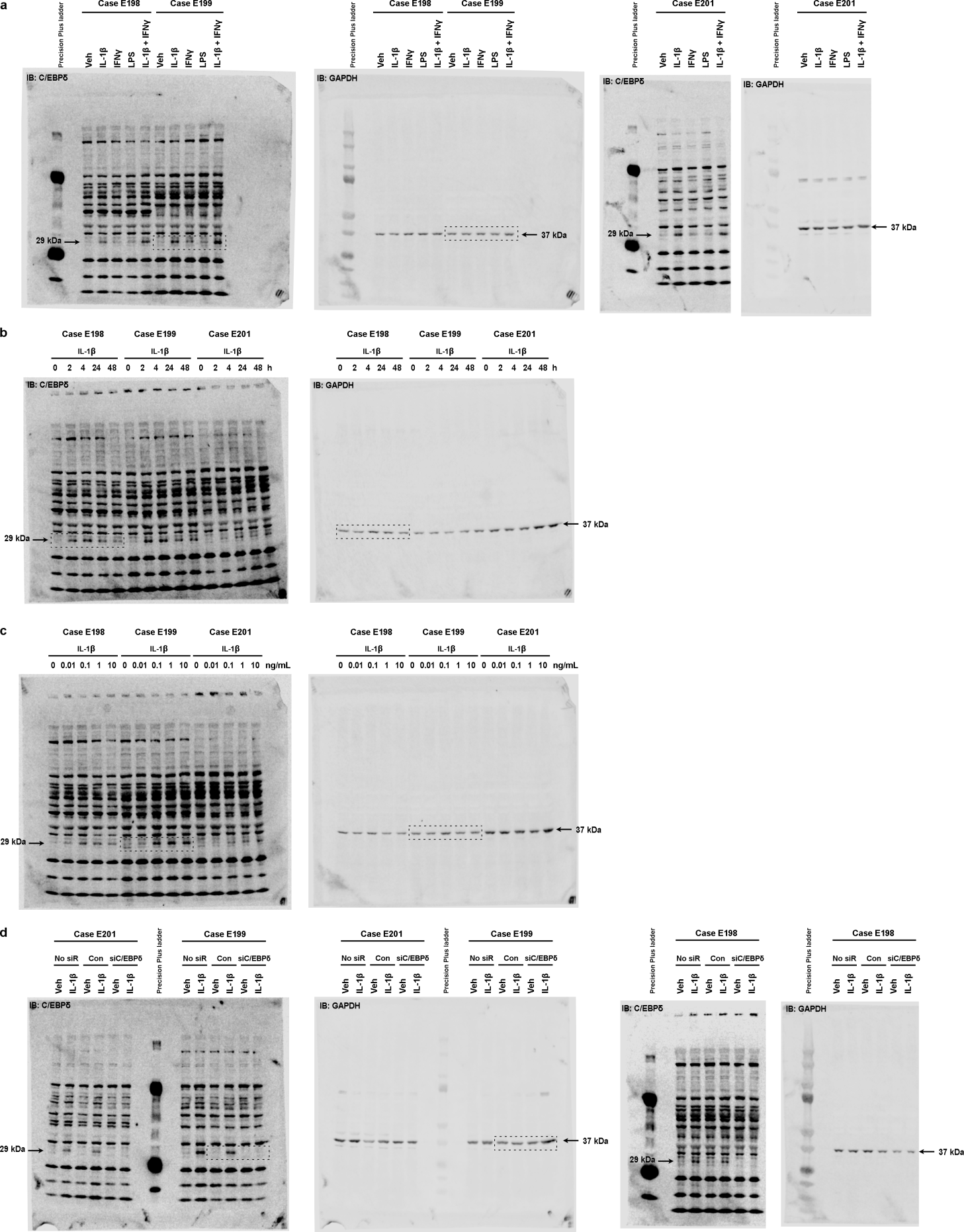


**Figure S2. Full length blots for western blot validation of C/EBPδ changes.** Human brain pericytes were treated as per details in corresponding figures (a-Figure 3, b,c-Figure 4 and d-Figure 5). Dotted lines represent cropped regions shown in Figures 3-5 with E198, E199 and E201 designating the three individual epilepsy cases used.
